# Developing a selective culturing approach for *Campylobacter hepaticus*

**DOI:** 10.1371/journal.pone.0302861

**Published:** 2024-05-31

**Authors:** Sheaaz G. J. Sakur, Sarah L. Williamson, Anthony Pavic, Yuanshuo K. Gao, Taha Harris, Michael Kotiw, Wendy Isabelle Muir, Peter John Groves

**Affiliations:** 1 School of Life and Environmental Sciences, Faculty of Science, The University of Sydney, Camden, New South Wales, Australia; 2 Birling Laboratories, Bringelly, New South Wales, Australia; 3 Sydney School of Veterinary Science, Faculty of Science, The University of Sydney, Camden, New South Wales, Australia; 4 School of Health and Wellbeing, University of Southern Queensland, Toowoomba, Australia; Beni Suef University Faculty of Veterinary Medicine, EGYPT

## Abstract

*Campylobacter hepaticus*, the causative agent of Spotty Liver Disease (SLD) is an important disease in cage-free egg producing chickens causing mortality and production drops. *C*. *hepaticus* is a slow growing *Campylobacter* easily overgrown by fecal bacteria. It is currently only reliably isolatable from bile samples. A selective media for isolation from feces or environment would assist diagnosis and impact assessment. Growth of five Australian *C*. *hepaticus* isolates was studied using Horse blood agar (HBA), sheep blood agar (SBA), Bolton, Preston and Brain Heart Infusion (BHI) base media. Blood and/or bile were added to Bolton, Preston and BHI medias. *C*. *jejuni* was used as a positive control. Plates were incubated in duplicate under microaerophilic conditions at 42°C for 10 days and examined at days 3–5 and 7–10 of incubation. Each isolate was examined for sensitivity to 14 antimicrobials using HBA sensitivity plates. Growth was inhibited by BHI and by added bile, while blood improved growth. Further replicates using SBA, HBA, Bolton and Preston media showed best growth on Bolton agar with blood. All five *C*. *hepaticus* isolates were resistant to trimethoprim and vancomycin, while four were also resistant to rifampicin and bacitracin. Media based upon Bolton plus blood supplemented with vancomycin and trimethoprim might be used as the most appropriate media for selective growth of *C*. *hepaticus*. The addition of bile to media for *C*. *hepaticus* isolation and growth will inhibit growth and is not advised.

## Introduction

Spotty Liver Disease (SLD) is an economically significant, acute infectious disease predominantly affecting free-range farmed layer hens. The aetiological agent was first identified as a *Campylobacter sp* in 2015 [1] and subsequently named as *Campylobacter hepaticus* in 2016 [2]. SLD is characterised by an acute onset in layer hens approaching peak lay and causing mortality rates and egg production losses as high as 10% and 25% respectively [3]. The current control methods for SLD include treatment with antibiotic (often chlortetracycline), increased biosecurity measures and feed additives [3–5]. However, the emergence of chlortetracycline-resistant strains (believed to be plasmid mediated) has been reported and is raising doubts regarding the continued efficacy of this control strategy [4]. Much of the challenge associated with SLD has arisen due to *C*. *hepaticus* being only recently confirmed as the aetiological agent. Consequently, much remains to be elucidated as to the pathophysiology and epidemiology of SLD [1, 2].

*C*. *hepaticus* has been demonstrated to be transmitted by the fecal-oral route, as confirmed through field studies and outcomes of an experimental infectious disease transmission model, as well as an environmental niche of the gastrointestinal tract (GIT), particularly the cecum [4, 6, 7]. Currently, *C*. *hepaticus* has only been cultured aseptically from the liver and bile of SLD infected chickens. The microbe is microaerophilic (5–10% v/v CO_2_ and O_2_) and thermotolerant (37–42°C) [3, 8, 9]. *C*. *hepaticus* differs to *C*. *jejuni* in culture isolation on solid agar media, with culture on *Campylobacter*-selective culturing media such as Skirrow and modified cefoperazone charcoal deoxycholate agar (mCCDA) being inhibitory. Consequently, general-purpose medium such as Horse Blood Agar (HBA) and Sheep Blood Agar (SBA) have been utilised during culturing [1, 5, 10, 11]. The difficulty associated with developing a selective culturing approach for *C*. *hepaticus* can be attributed to the bacterium’s fastidious nature, which readily causes cells to enter a state of dormancy upon exposure to stressors, such as prolonged exposure to atmospheric levels of oxygen [11, 12]. This is often regarded as a viable, but non-culturable state (VBNC). This change is often observed from its characteristic infective microscopic vibroid shape into a coccoid morphology when in the transformed VBNC state [13, 14].

The purpose of this study is to explore the use of various media compilations and antimicrobials to maximise isolation of *C*. *hepaticus* from animal sample. The outcomes may enhance our knowledge of the physiochemistry of the microbe and in particular the bacterial attributes that may play significant roles in environmental persistence and transmission. This study aimed to develop a selective and reliable culturing methodology for *C*. *hepaticus* that would enable the selective isolation of the bacterium from microbiologically complex sources such as its GIT niche and post outbreak-sourced faecal samples. By doing so, it would lead to a deeper understanding of the bacterium’s environmental behaviour, pathophysiology and the epidemiology of SLD. Understanding these factors will allow more targeted preventative measures such as appropriate antimicrobial choices and development of feed additives and vaccines, thus improving the welfare, productivity, and profitability of susceptible poultry farms.

## Materials and methods

### Isolates and culture conditions

Five disease outbreak derived C. *hepaticus* isolates were examined in the study (isolates denoted as WT, MV 3.1, MV 4.1, WIN 04–2 & #9 NT by the isolating laboratory) and were sourced from frozen stock cultures stored at -80°C at Birling Laboratories (Bringelly NSW, Australia). Isolates were obtained from clinical outbreaks of SLD on a variety of farms, and their corresponding year and number of passages are described in Table 1.

**Table 1:** *C*. *hepaticus* isolates from bile from clinical SLD cases examined.

Isolate identity	State of origin	Year obtained	Passages to storage
*WT*	New South Wales	2019	5
*MV 3*.*1*	New South Wales	2019	3
*MV 4*.*1*	New South Wales	2020	3
*WIN 04–2*	New South Wales	2020	5
*#9 NT*	Queensland	2019	6

All isolates were derived from bile samples, and stored in Bolton broth (Oxoid, Thermo Fisher Scientific Pty Ltd, Scoresby, Victoria, Australia), without supplement, containing 15% glycerol and stored at −80°C. Resuscitation of the isolates involved sub-culturing of frozen isolates onto ready-to-use sheep blood agar (SBA) plates (Edwards Group Pty Limited, Narellan, NSW, Australia) that had been pre-warmed at 42°C for 1.5 h. Cultures were incubated under microaerophilic conditions using BD GasPak^™^ EZ Campy gas packs (Becton Dickson Pty Ltd, North Ryde, NSW, Australia) at 42°C for 4–5 days until confluent growth was observed. The gas generating sachets were changed every third day, or every time the plates were taken out of the culture containers. Oxoid anaerobic colour change indicators (Thermo Fisher Scientific Pty Ltd, Scoresby, Victoria, Australia) were used to confirm the atmospheric conditions inside the incubation containers.

### Growth of *C*. *hepaticus* isolates on various media

Five basal media were evaluated for their ability to grow the *C*. *hepaticus* isolates: horse blood agar (HBA, Edwards Group Pty Limited, Narellan, NSW, Australia), sheep blood agar (SBA), Bolton, Preston and Brain Heart Infusion (BHI) agars. These five media were chosen based on published data that indicated proven capacity to culture *C*. *hepaticus* [1, 9, 10, 15], while Bolton and Preston broth media have been used to successfully culture *Campylobacter* spp. [16, 17]. While HBA and SBA were bought, Bolton, Preston and BHI agars were prepared non-supplemented from their respective dehydrated culture media (Oxoid^™^: Thermo Fisher Scientific Pty Ltd, Scoresby, Victoria, Australia). All basal media were prepared in accordance with the manufacturer’s instructions. Further modifications to these basal media, included the addition of defibrinated horse blood (Oxoid) at 5% and/ or bile salts (Oxoid) at 0.5%, resulting in a total of 14 media options (Table 2). All media were prepared and stored at 4°C with an expiry of 7 days. A sterility plate and a *C*. *jejuni* isolate were utilised as a negative and positive control respectively.

**Table 2:** Mean annular radii^\1^ (mm) from the CDS antimicrobial susceptibility test on the *C*. *hepaticus* isolates (WT, MV 3.1, MV 4.1, WIN 04–2, #9 NT).

Antimicrobials	Mean Annular Radii (mm)					P = ^ɫ^
	WT	WIN 04–2	#9 NT	MV 3.1	MV 4.1	
Amoxycillin (AML25)	19^A^^B^	18^B^	24^A^	17^B^	21^A^^B^	*0*.*01*
Neomycin (N30)	13	14	18	12	14	*0*.17*
Erythromycin (E5)	12^C^	14^B^^C^	22^A^	16^B^^C^	18^B^	*0*.003*
Tetracycline (TE30)	6^B^	28^A^	26^A^	3 ^B^	3 ^B^	*<0*.*001*
Trimethoprim (W5)	0	0	0	0	0	1.00
Colistin sulphate (CT10)	8^C^	13^B^	18^A^	11^B^^C^	11^B^^C^	*<0*.*001*
Cefotaxime (CTX5)	14	14	19	19	14	*0*.*07*
Vancomycin (VA5)	0	0	0	1	0	0.15
Rifampicin (RD1)	0 ^B^	1 ^B^	12^A^	0 ^B^	0 ^B^	*0*.007*
Nalidixic acid (NA30)	5 ^A^ ^B^	3 ^A^ ^B^	6^A^^B^	3 ^B^	4 ^A^	*0*.24*
Clindamycin (DA2)	16	14	30	16	13	*0*.*46*
Spectinomycin (SH25)	18^A^	18^A^	6^B^	17^A^	12^A^	*0*.*007*
Quinupristin (QD15)	6	4	3	3	4	*0*.*82*
Bacitracin (B10)	2	1	2	3	2	*0*.*79*

§P = probability differences due to chance, one-way ANOVA or Kruskal-Wallis ANOVA

* if variance not homogenous by Brown-Forsythe test

^ABCD^ = means within the same row without common superscripts isolates differ (P<0.05)

^\1^A mean annular radius <6mm is regarded as resistant (resistant results are underlined).

All the *C*. *hepaticus* isolates and the *C*. *jejuni* isolate, to be used as a positive control, were resuscitated and sub-cultured on SBA in duplicate as described above. Following detection of confluent growth, one of the two plates were harvested with a 10 μl loop and was aseptically inoculated into a pre-warmed 9 mL peptone dilutant 0.1% bottle to create bacterial suspensions. The bacterial suspensions were homogenised adjusting the suspension to a turbidity of 0.5–1 McFarland (approx. 1.5–3.0 x 10^8^ CFU / mL) determined with a VITEK^™^ colorimeter (Biomerieux Australia, Baulkham Hills, NSW, Australia). Once adjusted, the bacterial suspension for each isolate were respectively sub-cultured onto each of the various pre-warmed test media in duplicate using the Streak-Plate Method [18]. The struck media plates were then incubated as described above. The streak-plate method was adapted to quantify growth as the bacterium’s slow growing and spreading nature resulted in the inability to identify singular colony forming units with the spread plate method.

### Examination of cultures

**Fig 1:**
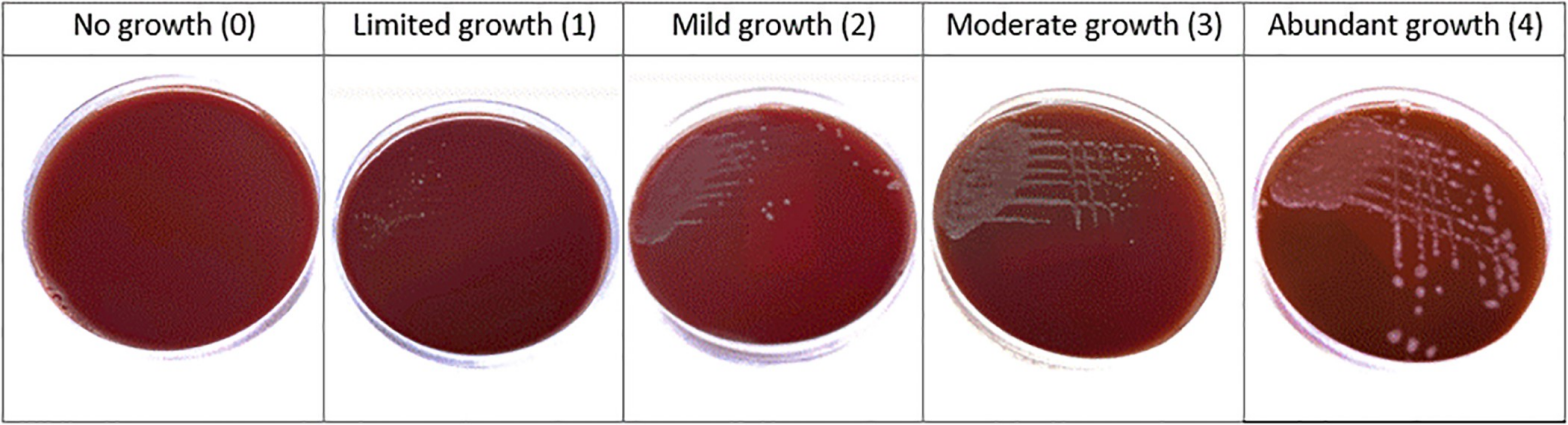
Depiction of categories used to quantify growth of *C*. *hepaticus* on solid media. *0 no growth, 1 limited growth, 2 mild growth, 3 moderate growth, 4 abundant growth.

The study comprised of a pilot study allowing the narrowing of the method in a follow up study which was repeated twice. Both studies involved a 10-day incubation, where the days of growth observed were reduced from days 3, 4, 7, 8, 9 and 10 in the pilot study to days 3, 5, 7 and 10 in the follow up study. A growth scale was formulated based on visual observation of growth and was developed at the end of the 10-day observation period of the pilot study after comparing among the various media. To quantify growth, a growth scale was developed based on observation of the amount of growth on various media (Fig 1). This consisted of five categories—abundant (scored as 4), moderate (3), mild (2), limited (1) or no growth (0). The follow up study involved the exclusion of BHI and bile and increasing replicates of each media from duplicates to triplicates.

On the tenth day, confirmatory tests were run for all the plates. As Skirrow media has been published as inhibitory of *C*. *hepaticus* [1], Skirrow plates (Edwards Group Pty Ltd, Narellan, NSW, Australia) were used as a negative control to aid as a confirmation that the growth being observed was *C*. *hepaticus*. A single colony was selected from each plate and struck onto Skirrow agar. A secondary confirmatory test by quantitative PCR (qPCR) was performed using primers as described by Van et al. [7] and the Type-it HRM PCR Kit, cat # 206546 (Qiagen, Chadstone, VIC, Australia) with a final primer concentration of 700nM. A crude bacterial suspension was made by suspending a 1uL loopful of suspect *C*. *hepaticus* in 500μl of molecular grade water (ThermoFisher Scientific Pty Ltd, Scoresby, Victoria, Australia), and vortexing before adding 5μl of this suspension to the PCR reaction. Cycling conditions were 95°C for 5min which will lyse the bacteria, followed by 40 cycles of 95°C for 10 sec, 57°C for 20sec and 72°C for 20 sec, with acquisition in the green channel during the 72°C extension step. A post amplification Melt step between 60–90°C (with 1°C increments) produced a specific Melt curve with a peak of 78.74±0.299°C confirming amplification of a specific PCR product and the presence of *C*. *hepaticus*. S1 Fig shows a melt curve analysis of a specific melt curve and quantitative analysis demonstrating product amplification.

### Antimicrobial susceptibility of *C*. *hepaticus* isolates

Fourteen antimicrobials were evaluated (Table 2) by disc diffusion methodology to determine the susceptibility of *C*. *hepaticus* isolates using Oxoid discs (Thermo Fisher Scientific Pty Ltd, Scoresby, Victoria, Australia) and HBA sensitivity plates (ThermoFisher Scientific Pty Ltd, Scoresby, Victoria, Australia). Three discs were loaded per plate, resulting in five plates per isolate in triplicate. A sterility plate and two plates of each isolate without antimicrobial discs were used as a negative and positive controls respectively.

All the *C*. *hepaticus* isolates were resuscitated as described above, with five SBA replicates being prepared for each isolate. After achieving confluent growth, bacterial suspensions were created as above and adjusted to a turbidity of 2 McFarland (approx. 6.0 x 10^8^ CFU/ mL) and spread plate inoculated on pre-warmed HBA sensitivity plates with 2.5 mL of the bacterial suspension. The plates were loaded with the antibiotic discs using an Oxoid^™^ Antimicrobial Susceptibility Disc Dispenser. The loaded plates were incubated for 4 days, and the annular radii were measured and recorded. The average annular radii among the replicates of the isolates were calculated and antimicrobials were termed resistant or sensitive according to CDS standard (radius of 6mm repeated a total of three times).

### Statistical analysis

For each isolate and media, growth was individually observed and categorised accordingly at day 10. For statistical analyses of growth, the nonparametric Kruskal-Wallis Analysis of Variance (ANOVA) was used to compare relative growth over the full ten days of culture for each isolate and within each of the media. Differences in antimicrobial susceptibility were identified using mean annular radii of zones of inhibition by one-way ANOVA unless variances were found not to be homogeneous by the Brown-Forsythe test, when Kruskal-Wallis ANOVA was used. The computerised statistical package STATISTICA v6 (StatSoft, Inc., 2003) [19] was used for analyses. All probability values <0.05 were considered significant.

## Results

### Growth of *C*. *hepaticus* on various media

All of the Bolton plus blood cultures were confirmed to be *C*. *hepaticus* by PCR from the after 10 days of growth. PCR confirmation was performed on colonies from non-selective media (i.e. HBA) and on colonies which had atypical colony morphologies to confirm that they were the target organism.

**Fig 2:**
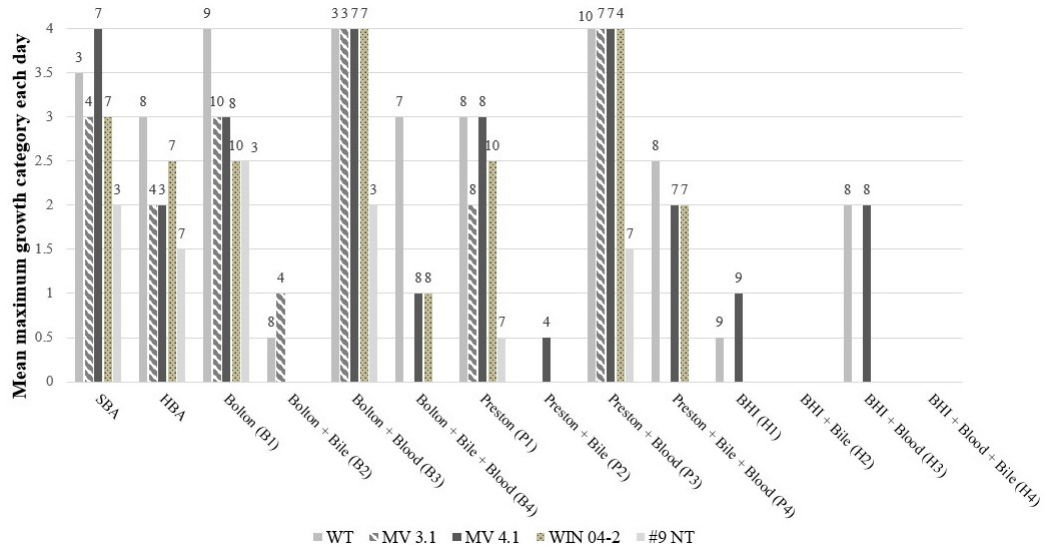
Maximum growth score (scale 0–4*, mean of 2 replicates) achieved by *C*. *hepaticus* isolates (WT, MV 3.1, MV 4.1, WIN 04–2 & #9 NT) on each media over a 10-day growth period. The first day at which maximum growth was observed is shown by the number above each bar. *0 no growth, 1 limited growth, 2 mild growth, 3 moderate growth, 4 abundant growth—see Fig 1.

**Fig 3:**
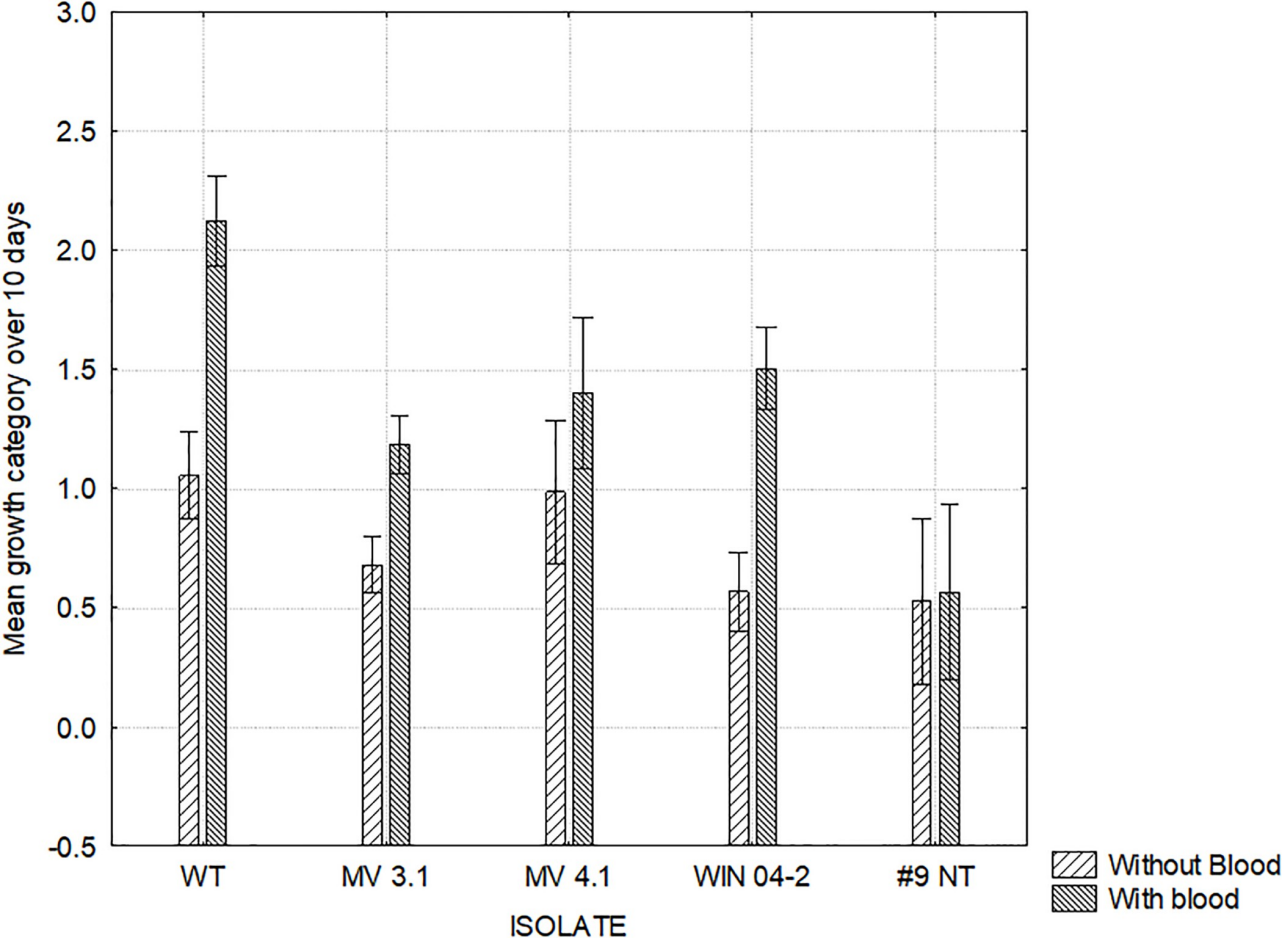
Growth of *C*. *hepaticus* isolates on Bolton, Preston and BHI media with or without blood. Mean growth categories: no growth (0), limited growth (1), mild growth (2), moderate growth (3) and abundant growth (4). ANOVA: F (4, 48) = 7.7085, p = .00007. Vertical bars denote 0.95 confidence intervals. A, B within the same isolate depicts means differ P<0.05.

**Fig 4:**
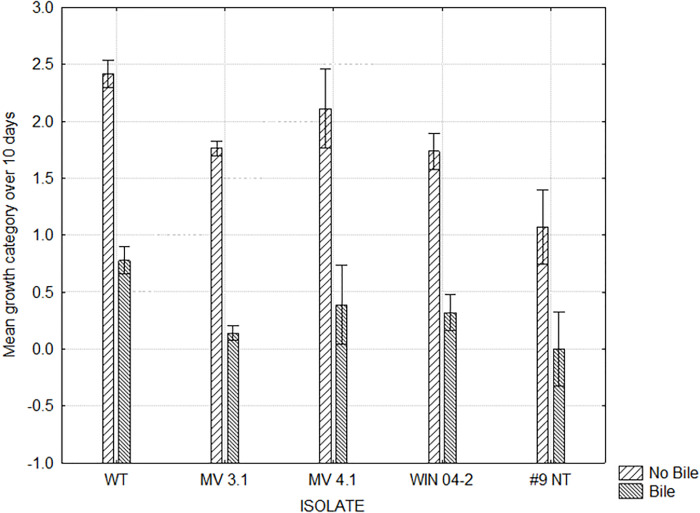
Growth of *C*. *hepaticus* isolates Bolton, Preston and BHI media with added Bile or no bile. Mean growth categories: no growth (0), limited growth (1), mild growth (2), moderate growth (3) and abundant growth (4). ANOVA: F (4, 48) = 3.0468, p = .02568. Vertical bars denote 0.95 confidence intervals. A, B within an isolate depicts means differ P<0.05.

**Fig 5:**
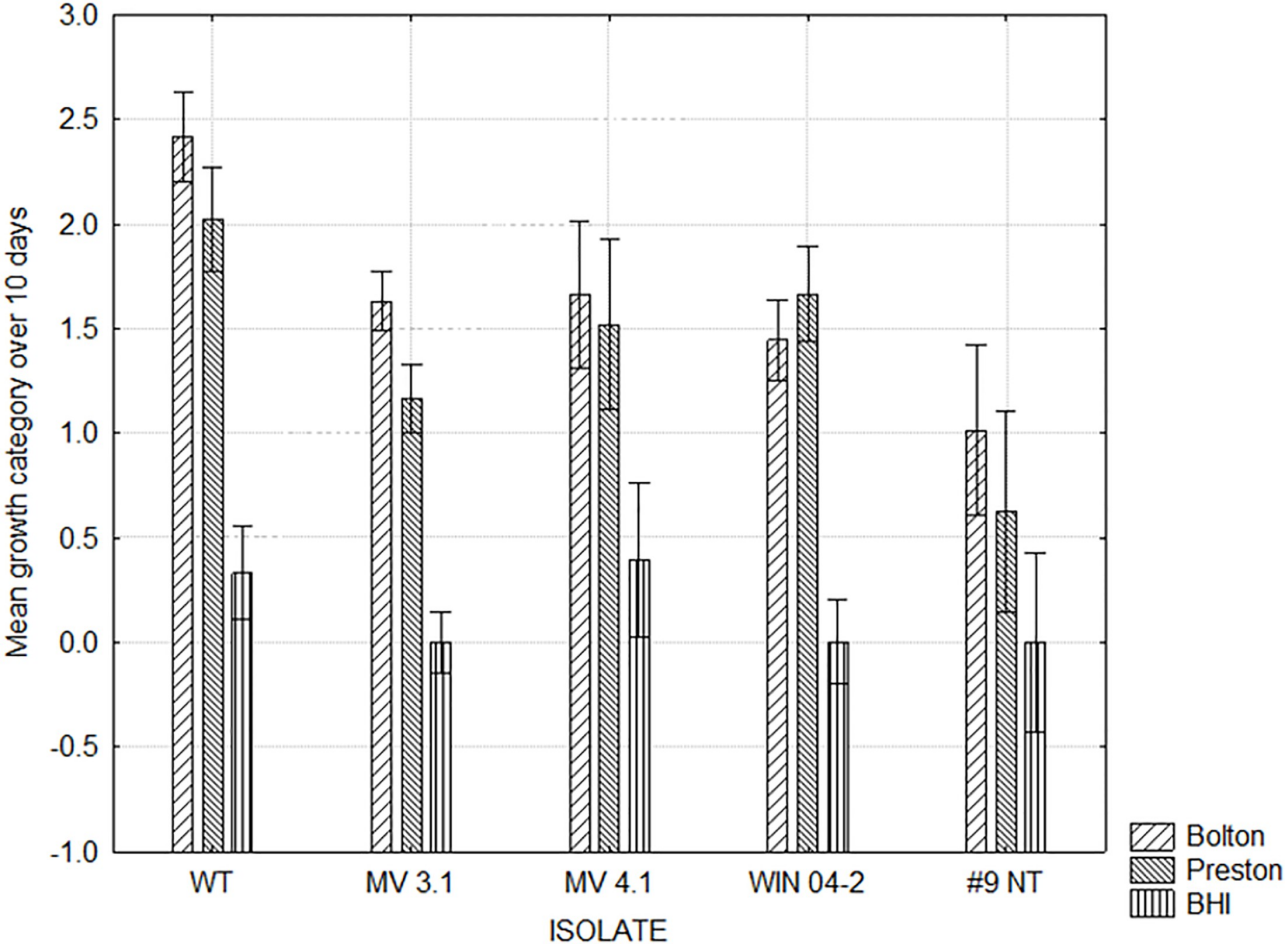
Growth of *C*. *hepaticus* isolates on Bolton, Preston or Brain Heart Infusion (BHI) media. Mean growth categories: no growth (0), limited growth (1), mild growth (2), moderate growth (3) and abundant growth (4). ANOVA: F (8, 48) = 4.3634, p = .00052. Vertical bars denote 0.95 confidence intervals. A, B, C depicts means within an isolate differ P<0.05.

**Fig 6:**
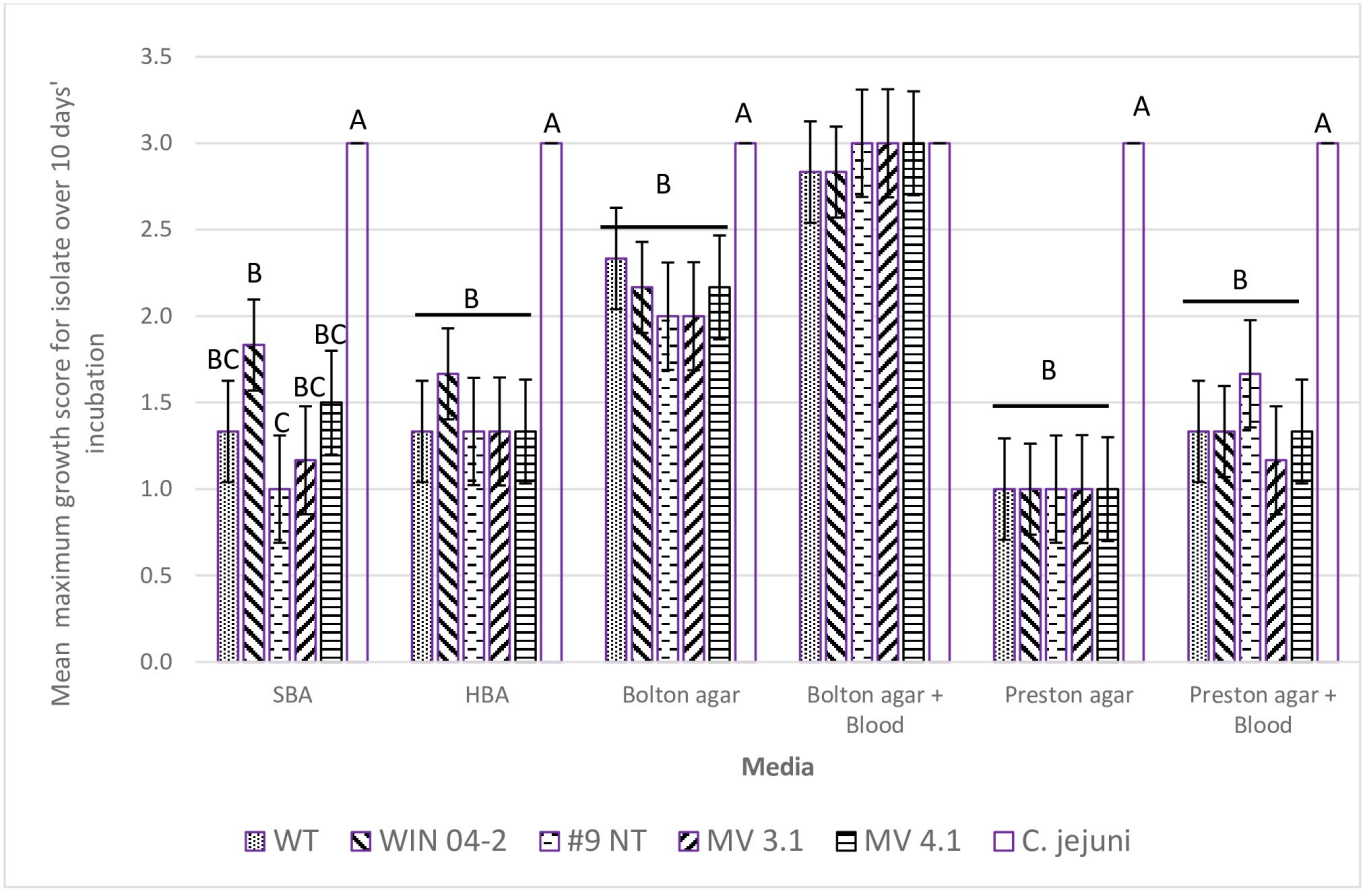
Maximum growth score in the follow up study (scale 0–4*, mean of 3 replicates) of *C*. *hepaticus* isolates (WT, MV 3.1, MV 4.1, WIN 04–2 & #9 NT) on various non-supplemented media over a 10-day growth period. Growth categories: no growth (0), limited growth (1), mild growth (2), moderate growth (3) and abundant growth (4). Vertical bars denote standard error. A, B, C—columns within each media with different superscripts differ (P<0.05, Kruskal-Wallis ANOVA).

Fig 2 shows the maximum growth score achieved and the day of culture on which the maximum growth was reached by each isolate on all 14 media combinations. This indicated that media containing blood encouraged growth compared to the basal media alone. This was seen particularly with isolates reaching their respective maximum growths more rapidly on Bolton and Blood and Bolton media (Fig 2). Figs 3 and 4 show the mean growth score achieved by each isolate across all media types with and without supplemental blood and bile respectively. While the supplementation of blood was able to significantly improve the growth of isolates WT, MV 3.1 and WIN 04–2, the growth of the #9 NT remained almost identical without blood (Fig 3). The supplementation of bile significantly reduced the growth of all five isolates (Fig 4). Fig 5 shows the mean growth achieved by each isolate on Bolton, Preston and BHI media (with and without supplements) over 10 days. The BHI media, regardless of supplementation, compared with Bolton and Preston media, supported only limited to no growth among the isolates regardless of the supplementation of blood and bile fluid (Fig 5). Despite displaying similar growth trends, isolates differed in their ability on the various media, with WT isolate exhibiting the most growth and the #9 NT isolate exhibiting the least amount of growth on the Bolton and Preston based media (Fig 5). The follow up study, where BHI and the addition of bile were eliminated, revealed similar results to the pilot study with isolates significantly achieving their maximum growth on Bolton and Blood, equivalent to the growth of *C*. *jejuni*. (Fig 6). The mean results of growth scores on each day of incubation for the pilot study and the follow up study are included in Supplementary information (Tables S1-S10 in S1 File).

A difference regarding colony morphology among the isolates was observed. On the non-bile containing media, growth of isolates WT, MV 3.1 and WIN 04–2 took the form of thinly spreading, cream coloured, irregular, flat films (mucoid growth) of variable sizes, while growth of isolates #9 NT and MV 4.1 took the form of focal, cream, low convex, smooth colonies (S2 Fig). Mucoid colonies have been assessed as the healthiest for the growth of the isolate (pers. comm. M. Kotiw). However, when bile was added to media, these phenotypic differences were not observed as all isolates displayed a common form of sparsely distributed, tiny, pinpoint colonies on the initial inoculum of the plate, or covered by a lawn growth. Previous studies have found pinpoint colonies to reflect stress on the bacteria (pers. comm. M. Kotiw). The *C*. *hepaticus* colonies on the bile containing medias were also observed during collection to have limited spreading and an adherence to the agar surface, which was not observed for *C*. *jejuni*.

### Antimicrobial susceptibility of *C*. *hepaticus* isolates

All five *C*. *hepaticus* isolates were resistant to trimethoprim and vancomycin antimicrobials (average annular radius of 0–1 mm—Table 2).

Resistance was observed against rifampicin in four out of five isolates (*WT*, *MV 3*.*1*, *MV 4*.*1 & WIN 04–2*) demonstrating resistance by an average annular radius of 0 mm except for *WIN 04–2* which averaged 1 mm. Isolate *# 9 NT* was markedly different to the other isolates, exhibiting susceptibility to rifampicin with an average annular radius of 12 mm (P< 0.007). Notable resistance against bacitracin was also observed in all of five isolates having an annular radius less than 6 mm. Significant isolates differences observed were notable with #9 NT exhibiting resistance to spectinomycin and susceptibility to rifampicin.

## Discussion

The study enabled the preliminary identification of media and additives that could allow the selective culture of *C*. *hepaticus* from complex bacterial sources, such as intestinal contents, feces or environmental sources.

### Inhibition of growth

The inhibition of growth associated with the supplementation of media with bile was an unexpected outcome as *C*. *hepaticus* has been successfully isolated from bile samples [4, 5, 20, 21]. This could suggest that the ability of *C*. *hepaticus* to tolerate bile may not be associated with the facilitation of growth but rather as a niche for survival. The ability to resist the antibacterial properties of bile provides enteric bacterial species such as *Campylobacter* spp not with just immunological avoidance, but with a selective advantage to outcompete neighbouring microflora present within the GIT [22, 23]. The *Campylobacter* genus does retain bile resistance mechanisms such as efflux pumps (e.g., CmeABC) [24, 25] and can alter virulence gene expression in the presence of bile (e.g. biofilm formation) [24, 25]. However, it is unknown if the lack of growth observed with *C*. *hepaticus* with increased bile concentrations was a result of cell death or cells entering a VBNC state. Furthermore, this lack of growth could also be due to the inability to simulate the exact *in vivo* conditions inside the chicken in the laboratory in-vitro. As there are other physiological parameters that can influence bile concentration and activity (e.g., presence of food or the type of diet) [20, 22, 26]. Hence, in future studies investigating the effects of bile on *C*. *hepaticus*, it is important that such conditions are taken into consideration.

Another unexpected finding was the limited growth detected in BHI basal media, as it had been previously reported as able to culture *C*. *hepaticus* [15]. The only growth observed was with two isolates (*WT & MV 4*.*1*) on BHI media that was supplemented with blood (Fig 2). It is unknown what component of the BHI media caused this inhibition. Studies have revealed that some bacterial species have a tolerance threshold for glucose, where high concentrations can result in antimicrobial-like effects, inhibiting bacterial growth [27, 28]. Generally, most *Campylobacter* spp. have been shown to be unable to catabolise glucose due to lacking key glycolytic enzymes and transporters for glycolytic pathways (e.g., Embden-Meyerhof-Parnas (EMP)) [29]. As such, it is possible that the addition of glucose in the BHI media and the inability for *C*. *hepaticus* to catabolise it could have resulted in high glucose concentrations exceeding its tolerance threshold, contributing to the inhibition observed. Ultimately it is suggested that BHI should not be used in attempts to culture *C*. *hepaticus*. Given only one published study [15] has demonstrated the ability for BHI to facilitate *C*. *hepaticus* growth, and this current study finding quite the opposite, it suggests that there could be possibly genetic differences among isolates. As such, more research needs to be conducted to identify or confirm possible limiting factors of the media (e.g., effect of various concentrations of glucose on growth) in relation to *C*. *hepaticus*.

### Promotion of growth

As field isolation of *C*. *hepaticus* can only be achieved on either HBA or SBA agar, it was assumed that the bacteria prefer blood in the culture media. As such, the supplementation of defibrinated horse blood to the media was expected to improve isolation and rate of growth of C. *hepaticus* isolates. This was shown to be the case in the use of Preston and Bolton based media (Fig 2). Nutrients within the cellular and plasma component of blood are known to promote growth of a variety of fastidious bacterial species by providing nutritional supplements source and in particular a source of iron [30]. Erythrocytes may protect bacteria from the toxic effects of oxygen derivatives and decrease the oxygen content in the culture, providing a more microaerobic environment [30, 31]. Therefore, considering the fastidious and microaerophilic nature of *C*. *hepaticus*, these properties of blood may explain why an improvement of growth was observed when it was added to media.

Supplementation of Bolton broth with blood resulted in the best growth outcome out of all 14 media. This is believed to be due Bolton’s non-supplemented base consisting of more growth enhancing components (i.e., sodium pyruvate, sodium metabisulphite and haemin) as compared to non-supplemented Preston. *Campylobacter* spp. have a limited ability to conserve energy for growth and maintenance being non-fermenters, thus the addition of sodium pyruvate provides an additional source of energy for bacterial metabolism and resuscitation [32–34]. Sodium metabisulphite acts similarly to blood as both an antioxidant and to decrease oxygen content [35, 36]. As Bolton is a blood free media, haemin acts as an iron source which is an essential nutrient for many cellular functions to promote growth and survival of many bacteria [36, 37] It is possible that Preston media could exhibit better growth than Bolton if the experiment were to be repeated with these/similar supplements added to Preston.

### Antimicrobial susceptibility profile

The results of utilisation of antimicrobials for selective enrichment of C. hepaticus growth are summarised in Table 2. All five isolates demonstrated resistance against trimethoprim, vancomycin, and bacitracin and four out of the five isolates (*WT*, *MV 3*.*1*, *MV 4*.*1* & *WIN 04–2*) demonstrated resistance against rifampicin. These results are consistent with published studies that have found *Campylobacter* spp. (e.g., *C*. *jejuni* and *C*. *coli*) to have intrinsic resistance against all four of these antimicrobials (trimethoprim, vancomycin, rifampicin, and bacitracin) and *C*. *hepaticus* may have similar characteristics [38–41]. Resistance against vancomycin and trimethoprim was also previously demonstrated by a study [15] that successfully isolated *C*. *hepaticus* using these antimicrobials. Other antimicrobials found to have intrinsic resistance (e.g., novobiocin, streptogramin B, etc.) against *Campylobacter* spp. should be tested for susceptibility in *C*. *hepaticus* [38, 39, 41].

As *C*. *hepaticus* demonstrated resistance to trimethoprim, vancomycin, rifampicin and bacitracin, these antimicrobials can be used to develop a selective media for *C*. *hepaticus* as it will allow growth of *C*. *hepaticus* while inhibiting other susceptible bacteria. However, to develop an effective selective media, the actions of the antimicrobial supplements should also be considered to understand which bacteria species these antimicrobials are selective against. These four antimicrobials have high activity against Gram-positive bacteria, and this is beneficial as the majority of bacteria species in the GIT of the chicken comprises of Gram-positive bacteria (e.g., *Clostridium*, *Enterococcus*, *Streptococcus*) [42–44]. In contrast, apart from trimethoprim, these antimicrobials have limited activity against Gram-negative bacteria, suggesting that the minority Gram-negative bacteria species present in the GIT may not be inhibited (e.g., *Escherichia coli* and *Bacteroides*) [42–44]. Theoretically, addition of these four antimicrobials will be successfully selective for *C*. *hepaticus*. However, contamination will almost always be inevitable during sampling and from the environment. As such, it may be useful for future studies to understand the effects of these four antimicrobials on Gram-negative bacteria of the GIT in chickens. However, studies have found that Gram negative bacteria such as *E*. *coli* may be susceptible to trimethoprim [45, 46]. Thus, the addition of these four antimicrobials may still be largely selective for *C*. *hepaticus*.

### Observed isolate differences

Each isolate was expected to conform to the various key trends observed, however there were multiple isolate differences that were observed throughout the study. It was assumed that by adjusting the bacterial suspensions to a turbidity of 0.5–1 McFarland (approx. 1.5–3.0 x 10^8^ CFU / ml), each plate would be inoculated and struck with same number of bacterial cells. This assumption could possibly rule out the difference among isolates being attributed to the difference in initial inoculum. However, compared to the pilot study where WT isolate was continually observed to have the best growth while #9 NT was observed to have the poorest growth, the follow up study showed #9 NT to have the best growth among the isolates. The remaining isolates *MV 3*.*1*, *WIN 04–2* and *MV 4*.*1* exhibited relatively similar growth in both studies. As this study was quantifying growth rates, it was also hypothesised that each isolate would have the same growth ability on the various media despite being obtained from different outbreaks. This hypothesis also applied to the antimicrobial susceptibility testing. However, isolates varied in the degree of susceptibility and resistance to the antimicrobials. This was observed with *#9 NT* isolate having a significantly different susceptibility to rifampicin as compared to the other four isolates. Additionally, colony morphology differences were observed between isolates, and this was also observed in a prior study [10]. These isolate differences could possibly result from unidentified differences such as genetic variation which is yet to be explored between strains of *C*. *hepaticus*. As such, the characterisation of the isolates (e.g., through clade identity by *Fla* typing or even whole genome sequencing) could be possible avenues for future research. Isolate #9 NT was the only isolate in this study not obtained from flocks in New South Wales, which may indicate differences due to geographic or epidemiological isolation.

Very recently a new *Campylobacter* species, newly named *C*. *bilis*, has been isolated from bile of chickens affected by SLD [12]. This species is genetically similar to *C*. *hepaticus* and is expected to be identified by the PCR used to detect it (R. Moore, pers. comm.) so it in light of that knowledge it is possible that the strains examined here could have included the new species. This needs to be further investigated but could be a possible source of variation observed in the present study.

#### Limitations

The current study did have limitations. The first limitation was the assumption that each plate was inoculated with the same initial concentration of bacterial cells. This was limiting, as there were various factors that could have introduced variation such as the homogeneity of the suspensions, amount of suspension attained on the loop and streaking pattern. Another limitation was the growth scale and the observational bias involved in characterising and interpretating it. Despite increasing replicates to triplicates in the follow up study to provide a more accurate measurements of growth, isolate differences became a limiting factor. Increasing isolate replication could better identify differences that were indeed due to isolate differences and not external factors such as inadequate streaking or incubation conditions.

This study used only a plate growth method as a measure of growth and a CFU/ mL method may provide more quantitative comparisons.

The current study serves as an avenue for the direction of further studies in the development of a selective media for isolation and culture of *C*. *hepaticus*. It is suggested that a selective media should be based upon Bolton media supplemented with blood and incorporating vancomycin and /or trimethoprim. A putative selective media needs to be further evaluated by challenging it with microflora common to the chicken GIT, including *E*. *coli*. Consideration of the use of rifampicin and bacitracin is also potentially useful but the variation in sensitivity of at least one *C*. *hepaticus* isolate in the current study would need further evaluation.

Only pure cultures were used in this study and further studies should evaluate ability of the suggested selective media to successfully isolate *C*. *hepaticus* from complex sources such as intestinal contents and feces.

## Conclusion

The current study revealed that a media based upon Bolton plus blood media supplemented with vancomycin and trimethoprim to be the most appropriate media for selective growth of *C*. *hepaticus*. The addition of bile to media for *C*. *hepaticus* isolation and growth will inhibit growth and is not advised.

## Supporting information

S1 Fig PCR conformation of *C*. *hepaticus*. A: Melt curve analysis showing a specific melt curve B: Quantitative analysis demonstrating product amplification.
(TIF)

S2 Fig Colony morphological differences. MV 4.1 isolate (A) exhibiting focal, cream, low convex, smooth colonies, and WT isolate (B) exhibiting cream, irregular flat films of variable sizes (mucoid) on Bolton agar supplemented with blood on day 3 of incubation.
(TIF)

S1 File Tables show data for relative growth of each isolate over a 10-day observation period for the pilot study and the follow up study. (DOCX)
