# Supplementary figures and images for: Developing a selective culturing approach for Campylobacter hepaticus
Source: PLoS One. 2024 May 31;19(5):e0302861. doi: 10.1371/journal.pone.0302861 (PMC11142446; doi:10.1371/journal.pone.0302861)

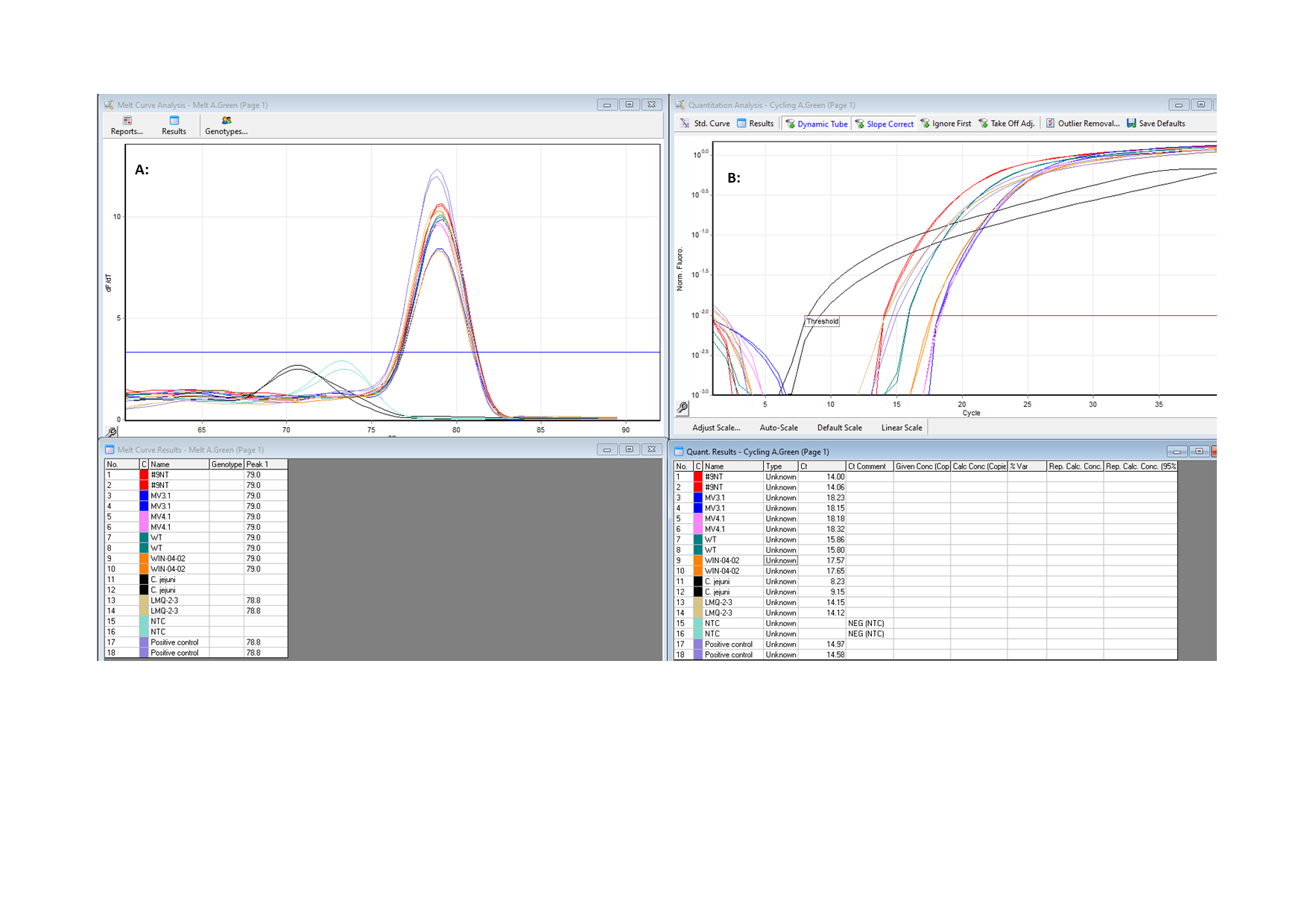

Supplement: S1 Fig — A: Melt curve analysis showing a specific melt curve B: Quantitative analysis demonstrating product amplification. (TIF) [file pone.0302861.s001.tif]

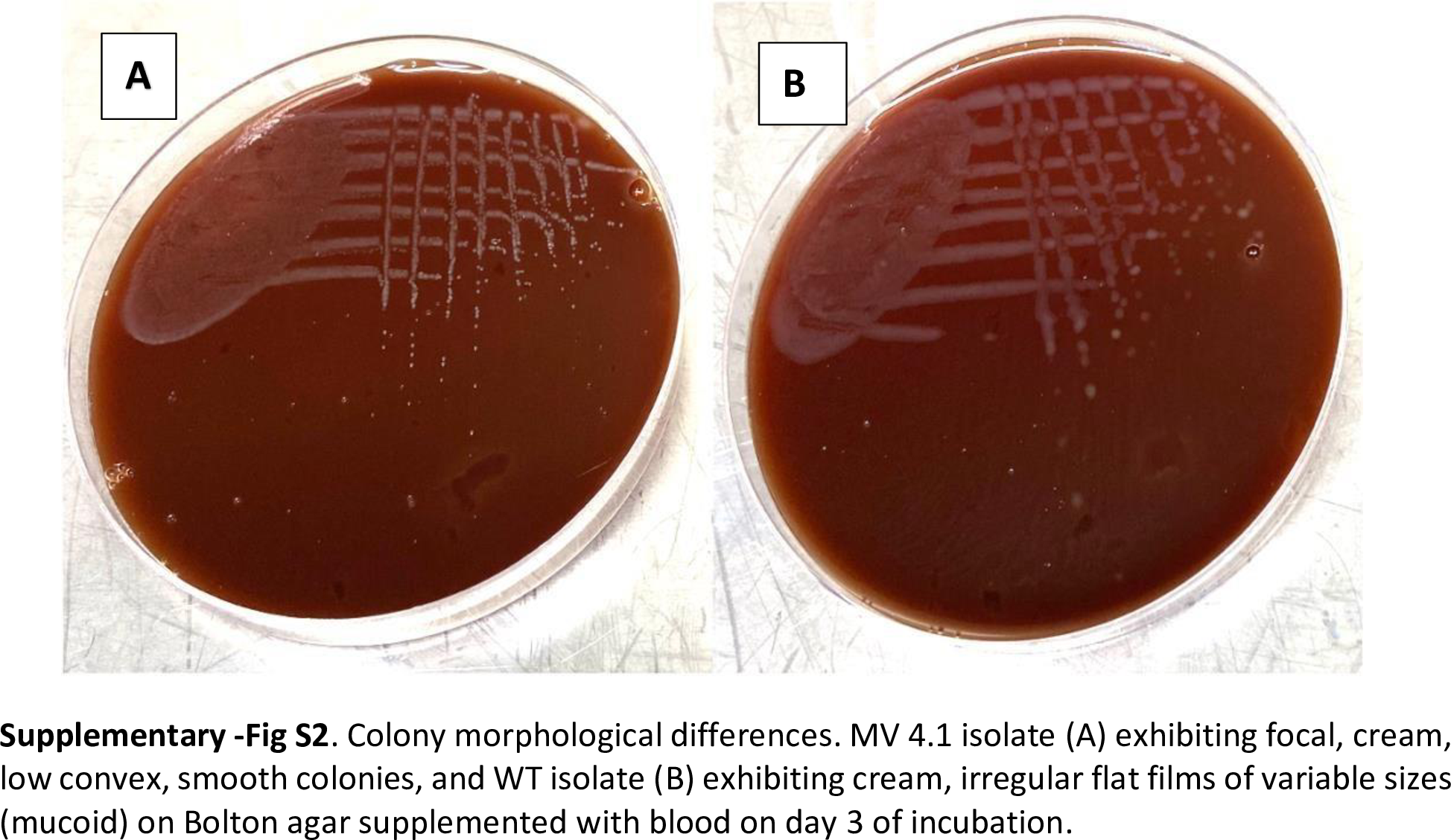

Supplement: S2 Fig — MV 4.1 isolate (A) exhibiting focal, cream, low convex, smooth colonies, and WT isolate (B) exhibiting cream, irregular flat films of variable sizes (mucoid) on Bolton agar supplemented with blood on day 3 of incubation. (TIF) [file pone.0302861.s002.tif]
