# Supplementary material for: Developing a selective culturing approach for Campylobacter hepaticus
Source: PLoS One. 2024 May 31;19(5):e0302861. doi: 10.1371/journal.pone.0302861 (PMC11142446; doi:10.1371/journal.pone.0302861)
Supplement: S1 File — (DOCX) [file pone.0302861.s003.docx]

**Supplementary information**

1. **Pilot Study**

Table S1. Relative growth score (scale 0-4*, mean of 2 replicates) of WT isolate on various media over a 10-day observation period

| **Media** | **Day 3** | **Day 4** | **Day 7** | **Day 8** | **Day 9** | **Day 10** |
| --- | --- | --- | --- | --- | --- | --- |
| SBA | 3.5 | 3.5 | 3.5 | 3.5 | 3.0Ϯ | 3.0Ϯ |
| HBA | 2.0 | 2.5 | 2.5 | 3.0 | 3.0 | 3.0 |
| Bolton Agar (B1) | 3.0 | 3.0 | 3.0 | 3.5 | 4.0 | 4.0 |
| Bolton + Bile Agar (B2) | 0.0 | 0.0 | 0.0 | 0.5 | 0.5 | 0.5 |
| Bolton + Blood Agar (B3) | 4.0 | 4.0 | 4.0 | 4.0 | 4.0 | 4.0 |
| Bolton + Blood + Bile Agar (B4) | 0.0 | 2.0 | 3.0 | 3.0 | 3.0 | 3.0 |
| Preston Agar (P1) | 2.0 | 2.0 | 2.0 | 3.0 | 3.0 | 3.0 |
| Preston + Bile Agar (P2) | 0.0 | 0.0 | 0.0 | 0.0 | 0.0 | 0.0 |
| Preston + Blood Agar (P3) | 2.0 | 3.0 | 3.5 | 3.5 | 3.5 | 4.0 |
| Preston + Bile + Blood Agar (P4) | 1.0 | 2.0 | 2.5 | 2.5 | 2.5 | 2.5 |
| BHI Agar (H1) | 0.0 | 0.0 | 0.0 | 0.0 | 0.0 | 0.0 |
| 0.0BHI + Bile Agar (H2) | 0.0 | 0.0 | 0.0 | 0..0 | 0.0 | 0.0 |
| BHI + Blood Agar (H3) | 0.0 | 0.0 | 1.0 | 2.0 | 2.0 | 2.0 |
| BHI + Bile + Blood Agar (H4) | 0.0 | 0.0 | 0.0 | 0.0 | 0.0 | 0.0 |
| * 0 no growth, 1 limited growth, 2 mild growth, 3 moderate growth, 4 abundant growth - see Figure 1.  Ϯ One replicate contaminated | | | | | | |

Table S2. Relative growth score (scale 0-4*, mean of 2 replicates) of MV 3-1 isolate on various media over a 10-day observation period

| **Media** | **Day 3** | **Day 4** | **Day 7** | **Day 8** | **Day 9** | **Day 10** |
| --- | --- | --- | --- | --- | --- | --- |
| SBA | 2.0 | 3.0 | 3.0 | 3.0 | 3.0 | 3.0 |
| HBA | 1.0 | 2.0 | 2.0 | 2.0 | 2.0 | 2.0 |
| Bolton Agar (B1) | 0.0 | 1.0 | 2.5 | 2.5 | 2.5 | 3.0 |
| Bolton + Bile Agar (B2) | 0.0 | 1.0 | 1.0 | 1.0 | 1.0 | 1.0 |
| Bolton + Blood Agar (B3) | 4.0 | 4.0Ϯ | 4.0Ϯ | 4.0Ϯ | 4.0Ϯ | 4.0Ϯ |
| Bolton + Blood + Bile Agar (B4) | 0.0 | 0.0 | 0.0 | 0.0 | 0.0 | 0.0 |
| Preston Agar (P1) | 0.0 | 1.0 | 1.0 | 2.0 | 2.0 | 2.0 |
| Preston + Bile Agar (P2) | 0.0 | 0.0 | 0.0 | 0.0 | 0.0 | 0.0 |
| Preston + Blood Agar (P3) | 2.0 | 2.0 | 4.0 | 4.0 | 4.0 | 4.0 |
| Preston + Bile + Blood Agar (P4) | 0.0 | 0.0 | 0.0 | 0.0 | 0.0 | 0.0 |
| BHI Agar (H1) | 0.0 | 0.0 | 0.0 | 0.0 | 0.0 | 0.0 |
| BHI + Bile Agar (H2) | 0.0 | 0.0 | 0.0 | 0.0 | 0.0 | 0.0 |
| BHI + Blood Agar (H3) | 0.0Ϯ | 0.0Ϯ | 0.0Ϯ | 0.0Ϯ | 0.0Ϯ | 0.0 |
| BHI + Bile + Blood Agar (H4) | 0.0 | 0.0 | 0.0 | 0.0 | 0.0 | 0.0Ϯ |
| * 0 no growth, 1 limited growth, 2 mild growth, 3 moderate growth, 4 abundant growth - see Figure 1.  Ϯ One replicate contaminated | | | | | | |

Table S3. Relative growth score (scale 0-4*, mean of 2 replicates) of MV 4.1 isolate on various media over a 10-day observation period

| **Media** | **Day 3** | **Day 4** | **Day 7** | **Day 8** | **Day 9** | **Day 10** |
| --- | --- | --- | --- | --- | --- | --- |
| SBA | 2.5 | 2.5 | 4.0 | 4.0 | 4.0 | 4.0 |
| HBA | 2.0 | 2.0 | 2.0 | 2.0 | 2.0 | 2.0 |
| Bolton Agar (B1) | 2.5 | 2.5 | 2.5 | 3.0 | 3.0 | 3.0 |
| Bolton + Bile Agar (B2) | 0.0 | 0.0 | 0.0 | 0.0 | 0.0 | 0.0 |
| Bolton + Blood Agar (B3) | 4.0 | 4.0Ϯ | 4.0Ϯ | 4.0Ϯ | 4.0Ϯ | 4.0Ϯ |
| Bolton + Blood + Bile Agar (B4) | 0.0 | 0.0 | 0.5 | 1.0 | 1.0 | 1.0 |
| Preston Agar (P1) | 1.0 | 2.0 | 2.0 | 3.0 | 3.0 | 3.0 |
| Preston + Bile Agar (P2) | 0.0 | 0.5 | 0.5 | 0.5 | 0.5 | 0.5 |
| Preston + Blood Agar (P3) | 2.0 | 2.0 | 4.0Ϯ | 4.0Ϯ | 4.0Ϯ | 4.0Ϯ |
| Preston + Bile + Blood Agar (P4) |  |  | 2.0 | 2.0 | 2.0 | 2.0 |
| BHI Agar (H1) | 0.0 | 0.0 | 0.0 | 0.5 | 1.0 | 0.0 |
| BHI + Bile Agar (H2) | 0.0 | 0.0 | 0.0 | 0.0 | 0.0 | 0.0 |
| BHI + Blood Agar (H3) | 0.0 | 0.0 | 1.0 | 2.0 | 2.0 | 2.0 |
| BHI + Bile + Blood Agar (H4) | 0.0 | 0.0 | 0.0 | 0.0 | 0.0 | 0.0 |
| * 0 no growth, 1 limited growth, 2 mild growth, 3 moderate growth, 4 abundant growth - see Figure 1.  Ϯ One replicate contaminated | | | | | | |

Table S4. Relative growth score (scale 0-4*, mean of 2 replicates) of WIN 04-2 isolate on various media over a 10-day observation period

| **Media** | **Day 3** | **Day 4** | **Day 7** | **Day 8** | **Day 9** | **Day 10** |
| --- | --- | --- | --- | --- | --- | --- |
| SBA | 2.0 | 2.5 | 3.0 | 3.0 | 3.0 | 3.0 |
| HBA | 1.0 | 2.0 | 2.5 | 2.5 | 2.5 | 2.5 |
| Bolton Agar (B1) | 1.0 | 1.0 | 2.0 | 2.0 | 2.0 | 2.5 |
| Bolton + Bile Agar (B2) | 0.0 | 0.0 | 0.0 | 0.0 | 0.0 | 0.0 |
| Bolton + Blood Agar (B3) | 2.0 | 2.0 | 4.0 | 4.0 | 4.0 | 4.0 |
| Bolton + Blood + Bile Agar (B4) | 0.0 | 0.0 | 0.5 | 1.0 | 1.0 | 1.0 |
| Preston Agar (P1) | 0.5 | 1.0 | 2.0 | 2.0 | 2.0 | 2.5 |
| Preston + Bile Agar (P2) | 0.0 | 0.0 | 0.0 | 0.0 | 0.0 | 0.0 |
| Preston + Blood Agar (P3) | 2.0 | 4.0 | 4.0 | 4.0 | 4.0 | 4.0 |
| Preston + Bile + Blood Agar (P4) | 0.0 | 0.0 | 2.0 | 2.0 | 2.0 | 2.0 |
| BHI Agar (H1) | 0.0 | 0.0 | 0.0 | 0.0 | 0.0 | 0.0 |
| BHI + Bile Agar (H2) | 0.0 | 0.0 | 0.0 | 0.0 | 0.0 | 0.0 |
| BHI + Blood Agar (H3) | 0.0 | 0.0 | 0.0 | 0.0 | 0.0 | 0.0 |
| BHI + Bile + Blood Agar (H4) | 0.0 | 0.0 | 0.0 | 0.0 | 0.0 | 0.0 |
| * 0 no growth, 1 limited growth, 2 mild growth, 3 moderate growth, 4 abundant growth - see Figure 1.  Ϯ One replicate contaminated | | | | | | |

Table S5. Relative growth score (scale 0-4*, mean of 2 replicates) of #9 N-T isolate on various media over a 10-day observation period

| **Media** | **Day 3** | **Day 4** | **Day 7** | **Day 8** | **Day 9** | **Day 10** |
| --- | --- | --- | --- | --- | --- | --- |
| SBA | 2.0 | 2.0 | 2.0 | 2.0 | 2.0 | 2.0Ϯ |
| HBA | 0.5 | 1.0 | 1.5 | 1.5 | 1.0Ϯ | 1.0Ϯ |
| Bolton Agar (B1) | 2.5 | 2.5 | 2.5 | 2.5 | 2.5 | 2.5 |
| Bolton + Bile Agar (B2) | 0.0 | 0.0 | 0.0 | 0.0 | 0.0 | 0.0 |
| Bolton + Blood Agar (B3) | 2.0 | 2.0 | 2.0 | 2.0 | 2.0 | 2.0 |
| Bolton + Blood + Bile Agar (B4) | 0.0 | 0.0 | 0.0 | 0.0 | 0.0 | 0.0 |
| Preston Agar (P1) | 0.0 | 0.0 | 1.0 | 1.0 | 1.0 | 1.0 |
| Preston + Bile Agar (P2) | 0.0 | 0.0 | 0.0 | 0.0 | 0.0 | 0.0 |
| Preston + Blood Agar (P3) | 0.5 | 1.0 | 1.5 | 1.5 | 1.5 | 1.5 |
| Preston + Bile + Blood Agar (P4) | 0.0 | 0.0 | 0.0 | 0.0 | 0.0 | 0.0 |
| BHI Agar (H1) | 0.0 | 0.0 | 0.0 | 0.0 | 0.0 | 0.0 |
| BHI + Bile Agar (H2) | 0.0 | 0.0 | 0.0 | 0.0 | 0.0 | 0.0 |
| BHI + Blood Agar (H3) | 0.0 | 0.0 | 0.0 | 0.0 | 0.0 | 0.0 |
| BHI + Bile + Blood Agar (H4) | 0.0 | 0.0 | 0.0 | 0.0 | 0.0 | 0.0 |
| * 0 no growth, 1 limited growth, 2 mild growth, 3 moderate growth, 4 abundant growth - see Figure 1.  Ϯ One replicate contaminated | | | | | | |

1. **Follow up study.**

Table S6. Relative growth score (scale 0-4*, mean of 3 replicates) of WT isolate on various media over a 10-day observation period in the follow up study.

| **Media** | **Day 3** | **Day 5** | **Day 7** | **Day 10** |
| --- | --- | --- | --- | --- |
| SBA | 1.0 | 1.3 | 1.3 | 1.3 |
| HBA | 1.0 | 1.0 | 1.0 | 1.3 |
| Bolton Agar (B1) | 2.0 | 2.7 | 2.7 | 2.7 |
| Bolton + Blood Agar (B3) | 3.0 | 3.0 | 3.0 | 3.0 |
| Preston Agar (P1) | 1.0 | 1.0 | 1.0 | 1.0 |
| Preston + Blood Agar (P3) | 1.0 | 1.3 | 1.3 | 1.3 |
| Skirrows | 0.0 | 1.0 | 1.0 | 1.0 |
| * 0 no growth, 1 limited growth, 2 mild growth, 3 moderate growth, 4 abundant growth - see Figure 1. | | | | |

Table S7. Relative growth score (scale 0-4*, mean of 3 replicates) of MV3.1 isolate on various media over a 10-day observation period in the follow up study.

| **Media** | **Day 3** | **Day 5** | **Day 7** | **Day 10** |
| --- | --- | --- | --- | --- |
| SBA | 1.0 | 1.3 | 1.3 | 1.3 |
| HBA | 1.0 | 1.0 | 1.7 | 1.7 |
| Bolton Agar (B1) | 1.0 | 2.0 | 2.0 | 2.0 |
| Bolton + Blood Agar (B3) | 2.0 | 2.3 | 3.0 | 3.0 |
| Preston Agar (P1) | 1.0 | 1.0 | 1.0 | 1.0 |
| Preston + Blood Agar (P3) | 1.0 | 1.0 | 1.3 | 1.3 |
| Skirrows | 0.0 | 1.0 | 1.0 | 1.0 |
| * 0 no growth, 1 limited growth, 2 mild growth, 3 moderate growth, 4 abundant growth - see Figure 1. | | | | |

Table S8. Relative growth score (scale 0-4*, mean of 3 replicates) of MV4.1 isolate on various media over a 10-day observation period in the follow up study.

| **Media** | **Day 3** | **Day 5** | **Day 7** | **Day 10** |
| --- | --- | --- | --- | --- |
| SBA | 1.3 | 1.7 | 1.7 | 1.7 |
| HBA | 1.0 | 1.3 | 1.3 | 1.3 |
| Bolton Agar (B1) | 1.3 | 2.3 | 2.3 | 2.3 |
| Bolton + Blood Agar (B3) | 2.0 | 3.0 | 3.0 | 3.0 |
| Preston Agar (P1) | 1.0 | 1.0 | 1.0 | 1.0 |
| Preston + Blood Agar (P3) | 1.3 | 1.3 | 1.3 | 1.7 |
| Skirrows | 0.0 | 1.0 | 1.0 | 1.0 |
| * 0 no growth, 1 limited growth, 2 mild growth, 3 moderate growth, 4 abundant growth - see Figure 1. | | | | |

Table S9. Relative growth score (scale 0-4*, mean of 3 replicates) of WIN 04-2 isolate on various media over a 10-day observation period in the follow up study.

| **Media** | **Day 3** | **Day 5** | **Day 7** | **Day 10** |
| --- | --- | --- | --- | --- |
| SBA | 1.3 | 1.3 | 1.3 | 1.7 |
| HBA | 1.0 | 1.0 | 1.0 | 1.3 |
| Bolton Agar (B1) | 1.3 | 2.0 | 2.3 | 2.3 |
| Bolton + Blood Agar (B3) | 2.0 | 3.0 | 3.0 | 3.0 |
| Preston Agar (P1) | 1.0 | 1.0 | 1.0 | 1.0 |
| Preston + Blood Agar (P3) | 1.0 | 1.0 | 1.3 | 1.3 |
| Skirrows | 0.0 | 1.0 | 1.0 | 1.0 |
| * 0 no growth, 1 limited growth, 2 mild growth, 3 moderate growth, 4 abundant growth - see Figure 1. | | | | |

Table S10. Relative growth score (scale 0-4*, mean of 3 replicates) of #9 N-T isolate on various media over a 10-day observation period in the follow up study.

| **Media** | **Day 3** | **Day 5** | **Day 7** | **Day 10** |
| --- | --- | --- | --- | --- |
| SBA | 1.0 | 1.0 | 1.0 | 1.0 |
| HBA | 1.0 | 1.3 | 1.3 | 1.3 |
| Bolton Agar (B1) | 1.3 | 2.0 | 2.0 | 2.0 |
| Bolton + Blood Agar (B3) | 2.0 | 3.0 | 3.0 | 3.0 |
| Preston Agar (P1) | 1.0 | 1.0 | 1.0 | 1.0 |
| Preston + Blood Agar (P3) | 1.3 | 1.7 | 1.7 | 2.0 |
| Skirrows | 0.0 | 0.0 | 0.0 | 0.0 |
| * 0 no growth, 1 limited growth, 2 mild growth, 3 moderate growth, 4 abundant growth - see Figure 1. | | | | |
